# Genomic and Functional Analysis of the Toxic Effect of Tachyplesin I on the Embryonic Development of Zebrafish

**DOI:** 10.1155/2014/454310

**Published:** 2014-04-29

**Authors:** Hongya Zhao, Jianguo Dai, Gang Jin

**Affiliations:** ^1^Industrial Center, Shenzhen Polytechnic, Shenzhen 518055, China; ^2^School of Applied Chemistry and Biotechnology, Shenzhen Polytechnic, Shenzhen 518055, China

## Abstract

Tachyplesin I (TP I) is an antimicrobial peptide isolated from the hemocytes of the horseshoe crab. With the developments of DNA microarray technology, the genetic analysis of the toxic effect of TP I on embryo was originally considered in our recent study. Based on our microarray data of the embryonic samples of zebrafish treated with the different doses of TP I, we performed a series of statistical data analyses to explore the toxic effect of TP I at the genomic level. In this paper, we first employed the hexaMplot to illustrate the continuous variation of the gene expressions of the embryonic cells treated with the different doses of TP I. The probabilistic model-based Hough transform was used to classify these differentially coexpressed genes of TP I on the zebrafish embryos. As a result, three line rays supported with the corresponding 174 genes were detected in our analysis. Some biological processes of the featured genes, such as antigen processing, nuclear chromatin, and structural constituent of eye lens, were significantly filtered with the smaller *P* values.

## 1. Introduction

Tachyplesin I (TP I) is originally isolated from the acid extracts of hemocytes of the horseshoe crab* Tachypleus tridentatus* in 1988 [1]. Since then, a series of biochemical analyses are made to gain sight into it. TP I consists of 17 amino acid residues, two disulfide bonds, and one unique *α*-arginine at the C terminal end and is characterized by a disulfide-stabilized *β*-sheet conformation. Among these 17 alpha amino acid residues, 4 cysteine residues constitute two disulfide bonds that contribute to the hemolytic ability of TP I in blood cells [2]. Furthermore, research has shown that TP I is active against both Gram-positive and Gram-negative bacteria [3], fungi [4], viruses [5], and cancer cells [6]. The peptide also interacts with DNA and inhibits the synthesis of macromolecules. Because of its potency and relatively small size, this peptide is a promising candidate of a novel alternative antibiotic in the pharmaceutical industry and animal and food industries. In comparison with the biochemical structure and mechanism of TP I, however, the literature on the toxicity of TP I is very limited, except its hemolytic ability.

In recent years the microarray technology is developed to be a powerful tool for investigation of functional genes that at present has become routine in many research laboratories [7–13]. To our best knowledge, microarray analysis for transcriptome changes of TP I has not been done. Therefore, the microarray experiment is originally designed to assess the toxic effect of TP I on embryo in our lab.

Despite a long tradition of using rats and mice to model human disease, several aspects of rodent biology limit their use in large-scale genetic and therapeutic drug screening programs. Currently, many researchers have sophisticatedly generated zebrafish (*Danio rerio*) models of a wide variety of human diseases. By contrast, zebrafish offers a model system that is ideally suited for large-scale microarray analysis. Zebrafish is a small vertebrate species, easily subjected to chemical mutagens and large numbers of mutant zebrafish. Given these advantages, zebrafish hold tremendous potential for the identification of toxic-causing and toxic-modifying genes [5, 6]. So the embryos of zebrafish were selected in our microarray experiment. The main aim of this study is to identify the functional coexpressed genes that show significant differential expression in the embryonic cells of zebrafish treated with the different doses of TP I. Obviously, the functional analysis of the toxic effect of TP I at genome level may reveal more advantages and disadvantages as a novel alternative antibiotic in the future.

The modified hexaMplot is employed to illustrate the gene expression alterations in our micoarray data analysis. The original hexaMplot is a two-dimensional representation of three kinds of intensities for assessing the drug effect [13]. Its coordinates represent the log ratios of intensity pairs: *x*_1_ = log⁡*E*_2_/*E*_1_ and *x*_2_ = log⁡*E*_1_/*E*_0_, where *E*_0_, *E*_1_, and *E*_2_ reflect the expression levels of genes in the normal, disease, and drug-treated samples, respectively. Note that genes appearing in the upper and lower half-plane of the hexaMplot are up- and downregulated, respectively, by the disease. Analogously, genes located in the left and right half-plane of the hexaMplot are up- and downregulated, respectively, by the drug treatment, compared with the disease sample. As a result, the slant axis *x*_2_ = −*x*_1_ is considered as *x*_3_ = log⁡*E*_2_/*E*_0_. Obviously, along the axis the expression levels of genes in the normal and drug-treated samples are the same.

Naturally the three axes and six regions in hexaMplot are meaningful for assessing drug effect [13]. Some methodologies are proposed to assess the drug effect based on the hexaMplot [13–15]. In comparison with the previous algorithms to consider the differently expressed genes only, the probabilistic model-based HT is proposed to address noise and quantization with the contribution of all assayed genes by posterior probabilities [15]. The performance of the algorithm is proved to be more robust and powerful. So we analyze our microarray data with the model-based HT algorithm.

Note that experiment design to analyze the toxic effect of the different doses of TP I is different from that to assess the drug. The gradual alterations of the gene expressions with the different doses are of our interest. So we first modified the axes of the original hexaMplot with *x*_1_ = log⁡*E*_1_/*E*_0_, *x*_2_ = log⁡*E*_2_/*E*_0_, and *x*_3_ = log⁡*E*_2_/*E*_1_, where *E*_0_, *E*_1_, and *E*_2_ reflect expression levels of the genes in the normal and 1.5 ug/mL and 2.5 ug/mL treated samples, respectively. Analogously, the corresponding axes and quadrants also show the significant meanings to assess the toxic effect on assayed genes treated with the different doses of TP I.

Furthermore, the model-based HT in [15] was used to identify the functional genome groups along the line rays. Three line rays supported with the corresponding 174 genes are detected in our microarray data. The feature genes identified on the same line ray of hexaMplot may show the similar expression patterns in varying the doses of TP I. The functional groups of these genes, such as antigen processing, nuclear chromatin, and structural constituent of eye lens, show coherent biological function with high significance as detected by gene ontology analysis.

The paper has the following organization: after introducing our microarray experiment in Section 2, we apply the modified hexaMplot to demonstrate the gene alteration among the embryonic samples with the different doses and model-based HT to identify the function gene groups in Section 3. The conclusion and discussion are summarized in Section 4.

## 2. Methodology

In this section, the microarray experiment to assess the toxic effect of TP I on the embryonic development of zebrafish is described in Section 2.1. Then, the statistical tools to analyze the microarray data are mentioned in Section 2.2.

### 2.1. Microarray Gene Expression Experiment

Considering the great advantages of zebrafish in model system, we select the zebrafish model for exploring the potential developmental toxicity of TP I in our microarray experiment. TP I was synthesized by Shenzhen Han Yu Pharmaceutical Co., Ltd. (purity, >95.6%) according to the sequence reported by Nakamura et al. (1988) [1]. Wild-type TU of zebrafish were obtained from the Peking University College of Chemical Biology and Biotechnology.

For the acute toxicity test, zebrafish embryos of 3 hours postfertilization (hpf) at three stages of embryonic development were exposed to TP I in a 6-well plate up to 24 hpf. The embryonic development of zebrafish is commonly divided into seven stages, the zygote (0–0.75 hpf), cleavage (0.75–2.25 hpf), blastula (2.125–5.25 hpf), gastrula (5.25–10 hpf), segmentation (10–24 hpf), pharyngula (24–48 hpf), and hatching (48–72 hpf) periods. And we also found that the abnormal morphology of 3 hpf embryos treated with TP I appeared at 24 hpf. Therefore, we collected 20 hpf embryos to extract total RNA for gene microarray analysis.

**Figure 1:**
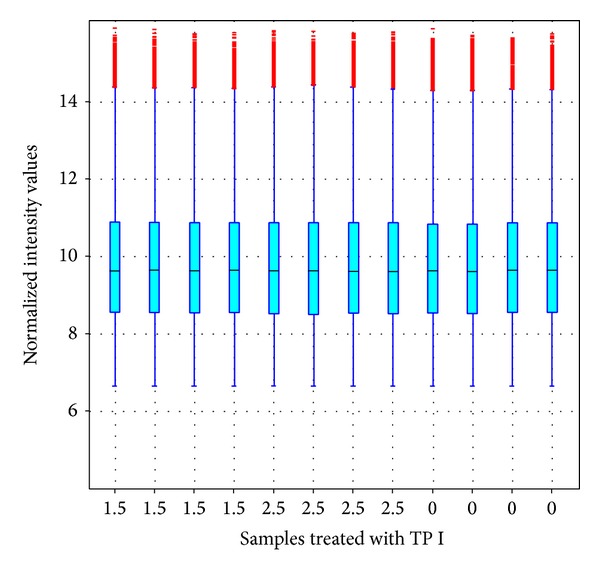
The boxplot of the normalized expression data of zebrafish embryonic samples treated with 1.5- and 2.5-*μ*g/mL TP I and blank samples. Four replicates were used for each sample.

The concentrations used were as follows: 0, 1.5, and 2.5 *μ*g/mL. Four replicates were made for each concentration, and each replicate consisted of three wells. Each well contained 10 mL of treatment solution and 50 viable embryos. At 20 hpf, 50 abnormal embryos were selected from each replicate to extract total RNA for gene microarray. Four gene chips were used for each concentration. The NimbleGen array used interrogates 38,489 transcripts from Ensembl build Zv7 with 3 probes per transcript. The raw intensities were normalized in RMA method by NimbleScan v2.5 and the data quality was assessed by boxplot and scatter plot. The raw intensities were normalized in RMA method by NimbleScan v2.5 and the data quality was assessed by boxplot and scatter plot, as demonstrated in Figure 1. All in all, the expression values of 26272 transcript sequences in 12 samples of zebra embryo treated with the different doses of TP I are recorded.

### 2.2. HexaMplot and the Probabilistic Model-Based Hough Transform

Considering the different doses of TP I with 0, 1.5 *μ*g/mL, and 2.5 *μ*g/mL, we employed the probabilistic model-based HT in the modified hexaMplot to assess the toxic effect of TP I on the embryonic development of zebrafish. The original hexaMplot is a two-dimensional representation of R, G, and B intensities and proposed to assess the drug effect on assayed genes in [7]. HexaMplot provides a simple, intuitive, and efficient tool for assessing drug effect.

**Figure 2:**
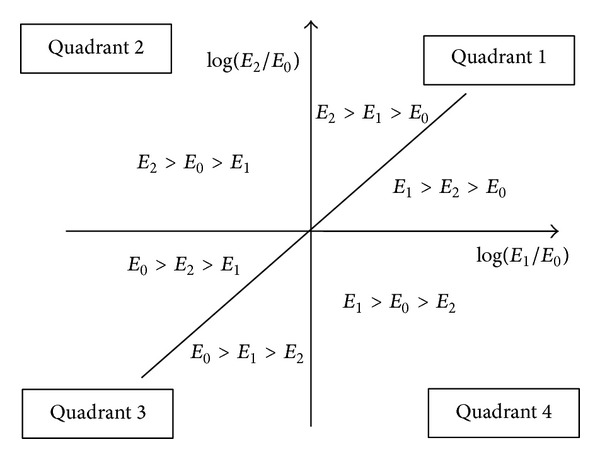
The modified layout of the hexaMplot to assess the toxic effect.

Similarly, we modified the raw hexaMplot with *x*_1_ = log⁡*E*_1_/*E*_0_ and *x*_2_ = log⁡*E*_2_/*E*_0_, where *E*_0_, *E*_1_, and *E*_2_ reflect expression levels of the genes in the normal and 1.5 and 2.5 ug/mL treated samples, respectively. As demonstrated in Figure 2, the genes appearing in the upper and lower half-plane of the modified hexaMplot are up- and downregulated, respectively, by the 2.5 ug/mL TP I. Analogously, genes located in the left and right half-plane are up- and downregulated, respectively, by the treatment with 1.5 ug/mL TP I. Also note that along the slant axis *x*_2_ = *x*_1_, we have log⁡*E*_2_ = log⁡*E*_1_, meaning that the expression levels of genes in the 1.5 and 2.5 ug/mL treated samples are the same. Generally, the researchers would like to explore gradual alternation of assayed genes in varying the doses of TP I. The gene representation clustering the slant axis may imply that they are not sensitive to change of the doses of TP I.

Comparatively, the genes in quadrant 2 (Q2) and Q4 may reverse their expression patterns, either further enhancing upregulation or suppressing the downregulation of the gene by the adding dose. Generally, the three axes and six regions in the modified hexaMplot show intuitively the gradual change of gene expression to assess the toxic effect of TP I with the different doses.

Based on the intuitive illustration of hexaMplot, some statistical methods were proposed to identify the functional genes [13–15]. The probabilistic model-based HT was proposed in [15] to detect groups of genes with similar expression patterns with different doses of TP I. Each such group is aligned along a line ray starting in the hexaMplot or origin. The direction of the ray signifies whether the addition of TP I dose has positive or negative effect on expression of the group of genes, while the angle measures the effect level [7]. The lines were detected through HT. As discussed in [15], the model was improved by formulating the Bayesian probabilistic models with Gaussian kernel. Given an observation vector *E*, the posterior probability of parameter (*α*, *r*) is given by

(1) $$\begin{array}{c} p\left(\alpha ,r\;|\;E\right)=\frac{p\left(E\;|\;\alpha ,r\right)p\left(\alpha ,r\right)}{\int_{\Omega }p\left(E\;|\;\alpha^{\prime },r^{\prime }\right)p\left(\alpha^{\prime },r^{\prime }\right)d\alpha^{\prime }dr^{\prime }}, \end{array}$$

where *α* and *r* are the angle and distance of the point on the line from the origin and *Ω* is the range space of the two parameters. We are interested in the data points aligned along the line passing through the origin. So *r* can be integrated and then we obtain the probability of support for the angle *α* given the observation *p*(*α* | *E*). Given a set of observations and the corresponding Gaussian support kernel, the posterior probability of the set of selected points can be calculated to assess how much the set supports the line ray.

The probabilistic model showed some advantages in detecting the lines and their supporting gene points. First all assayed genes are considered in the algorithm instead of the differentially expressed genes only. Second the probabilistic model explicitly takes into account the size and the negatively correlated nature of the noise associated with hexaMplot gene representations. Third, both the strength of association of individual genes with a particular group (line ray in hexaMplot) and the support for the group by the selected genes can be quantified in a principled manner through posterior probabilities over the line angles [15].

## 3. Results 

### 3.1. The Identification of Significant Genes

With the microarray data matrix of 26272 rows and 12 columns, the direct 2-fold change with *P* < 0.05 in *t*-test was used to identify the significant genes. Obviously, the traditional *t*-test method can only compare between the treatment and control groups. There are two treatment groups in our microarray experiment. So the three groups of *t*-test between any two groups were made. First we compared the samples treated with 1.5 *μ*g/mL TP I and the blank groups. And 212 differentially expressed genes were identified, in which there were 102 upregulated genes and 110 downregulated.

Similarly, 307 significant genes were identified by comparing the expression data between 2.5 *μ*g/mL TP I treatment group and blank group. And 147 genes were upregulated and 260 were downregulated. We also detected the differentially expressed genes between the two groups, respectively, treated with 2.5 and 1.5 *μ*g/mL TP I. 111 upregulated and 245 downregulated genes are identified.

Among all of the differentially expressed genes, 51%, 34%, and 11% were associated with the biological process, molecular function, and cellular components in the analysis of gene ontology, respectively. We found that most of the identified genes are related to the Jak/STAT signaling pathway, adherent junction signaling pathway, and tight junction signaling pathway.

Furthermore, we screened a series of significant genes related to the development of zebrafish including Ntl (no tail) and Tbx24 (T-box 24) (related to body axis), Pes (pescadillo) (related to liver), and vascular endothelial growth factor- (VEGF-) Ab1 (related to vasculature). Microarray analysis showed that the expressions of the development-related differential expression genes CYP11A1, Pes, Ntl, VEGF-Ab1, and Tbx24 significantly changed. The findings suggest that TP I interfered with the normal embryonic development of zebrafish.

VEGF is a major vasculogenic and angiogenic factor in embryonic vessel development. It is involved in many biological processes, including the growth and differentiation of vein cells, the growth of endothelial cells, and the permeability of vessels. For zebrafish, VEGF-A and Flk-1 play a crucial role in the angiogenesis of axes and intersegments. Morphological analysis has detected the significant absence of both axial and intersegmental vasculature in zebrafish with downregulated VEGF [16, 17].

CYP11A1 (cytochrome P450 subfamily XIA polypeptide 1), a member of the CYP450 family, which is related to the metabolism of xenobiotics, is expressed in the yolk syncytial layer during early embryogenesis [18, 19]. Therefore, significant expression of CYP11A1 may generate pericardial edema. In the present study, VEGF and CYP11A1 were significantly down- and upregulated, respectively. So it may be concluded that they are related to pericardial edema and abnormalities in the intersegmental vasculature.

T-box transcription factors are a large family of transcriptional regulators involved in many aspects of embryonic development, such as the development of neural tubes and somites [20–26]. Ntl and Tbx24, which are zebrafish T-box genes, are required for the development of the trunk, notochord and tail mesoderm, and medial floor plate [21–23]. During the embryogenesis of zebrafish, Ntl and Tbx24 are located in and derived from notochord cells and somites, respectively [25]. The research has shown that inhibiting Ntl and Tbx24 causes spinal flexion and somite defects [26]. Our microarray analysis also showed that Ntl and Tbx24 were significantly down- and upregulated. We conclude that these transcription factors may cause spinal flexion.

### 3.2. Genomic and Functional Analysis of the Toxic Effect of TP I

**Figure 3:**
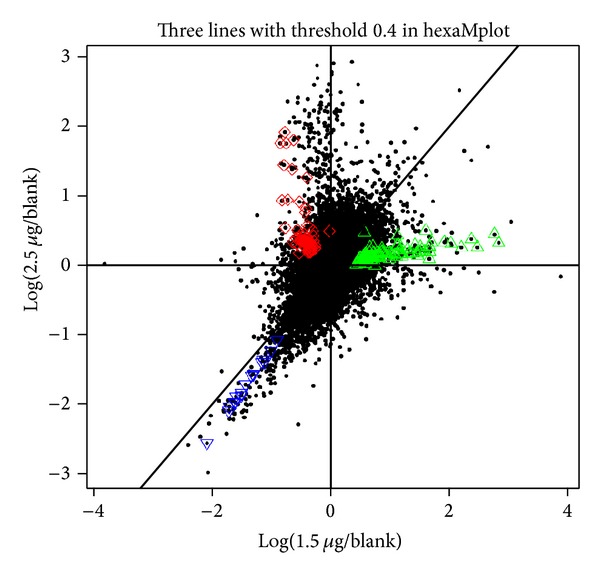
Detected line rays (solid bold lines) for sigma = 0.04 and selected points supporting the three lines.

In this subsection, we applied the modified hexaMplot and probabilistic model-based HT to our microarray data to assess the toxic effect of TP I on the embryonic development of zebrafish. There are 26272 spots assayed in 12 microarrays obtained in four repeats of the normal and 1.5 ug/mL and 2.5 ug/mL treated embryonic samples. The original microarray data were normalized using LIMMA algorithms between and within microarrays [8]. The modified hexaMplot of the normalized data is shown in Figure 3. Three significant lines can be detected with the Bayesian probabilistic model. The optimal threshold is set to *σ* = 0.04 according to the algorithm [15]. And the corresponding gene points supporting the lines are marked with red, green, and blue in Figure 3.

In comparison with the layout of Figure 2, we can infer some conclusion about the toxic effect of TP I with different doses. In Q1 of Figure 2, 93 green points were identified to support one line ray. And the angle *α* of the line ray in Q1 is small. In other words, the 93 genes that supported the line are greatly suppressed from the upregulation to normal with the increase of dose of TP I. The group of 64 genes in Q2 shows the reverse trend of expression; that is, the downregulation in the samples treated with 1.5 ug/mL is enhanced to upregulation with 2.5 ug/mL. As to the 17 blue points in Q3, the downregulated genes are further suppressed with adding the dose of TP I. But the angle bias from axis *x*_2_ = *x*_1_ is very small. So the alternation of downregulation is not significant.

It is well known that coexpressed genes, not one gene, are involved in the biological function together. So we investigated the biological meaning of the three detected groups of genes (the points supporting the line rays in Figure 3) with gene ontology (GO) framework [27]. The results of GO analysis are summarized in Table 1. The table has the following organization: representative GO terms and its biological meaning for genes are listed in the first and second column. For each GO term, we report four terms of enrichment in the last column, including the total number of genes (*N*), the number of genes annotated to the GO term (*B*), the number of genes from our assayed gene annotated to it (*n*), and the number of genes in the intersection (*b*). The corresponding *P* values of the GO terms enriched were computed according to the following hypergeometric distribution and the values were listed in the third column of Table 1. Consider

(2) $$\begin{array}{c} P\;\text{value}=\overset{B}{\underset{j=b}{\sum }}\frac{\left(\begin{array}{c} n\\ j \end{array}\right)\left(\begin{array}{c} N-n\\ B-j \end{array}\right)}{\left(\begin{array}{c} N\\ B \end{array}\right)}. \end{array}$$

According to the biological function in Table 1, the 93 genes on the green line in Q1 are mainly involved in the antigen processing and presentation of endogenous peptide antigen. And their upregulation of the gene group is a little suppressed with adding dose of TP I from 1.5 to 2.5 ug/mL. We may conclude that the varying doses of TP I show little effect on the antigen process.

Similarly, the 64 genes of the red line in Q2 are related to the chromatin, WINAC complex, or DNA-directed RNA polymerase II, holoenzyme. The adding dose can reverse the gene expression pattern from downregulation to upregulation.

As mentioned in the previous literature of TP I, the peptide is very active against both Gram-positive and Gram-negative bacteria, fungi, viruses, and cancer cells and can interact with DNA and inhibit the synthesis of macromolecules. Our result may further show the biological mechanism in genome level. The 17 genes in Q3 are of interest.

We found that these genes may play an important role in the structural constituent of eye lens. And the expression of downregulated genes is further enhanced with the increasing dose of TP I. It may be concluded that TP I shows the toxic side effect on eye lens and this effect is strengthened with the adding dose. The result may discover some biological mechanism of TP I in genome level. Of course, more experiments are required to be performed to gain insight into the toxic effect of TP I.

## 4. Conclusion and Discussion

The advantages of tachyplesin I as a novel antimicrobial peptide are appearing with the insight of its biochemical mechanism of strong antimicrobial and anticancer activity. We originally focused on the genetic analysis of TP I. A series of microarray experiments are performed in our research. In this paper, the toxic effect of TP I on the embryonic development of zebrafish was assessed on the genome level. The hexaMplot was used to illustrate the gene expressions with the varying doses of TP I. The probabilistic model-based Hough transform (HT) was used to classify these coexpressed genes.

In our analysis, three line rays supported with the corresponding 174 genes were detected. The three groups of genes were classified into the coherent GO terms with the high significance as detected by gene ontology analysis. The GO functional groups of these genes, such as antigen processing, nuclear chromatin, and structural constituent of eye lens, may explore the biological mechanism of TP I in genome level. In particular we found that TP I shows the toxic side effect on eye lens and this effect was strengthened with the adding dose, which was of interest in the literature of TP I and provided a new direction for our further research.

## Figures and Tables

**Table 1:** The toxic effect on the embryonic development of zebrafish.

Line	GO term	Description	*P* value	Enrichment (*N*, *B*, *n*, *b*)
1	GO:0002483	Antigen processing and presentation of endogenous peptide antigen	3.99*E*− 5	178.99 (6712, 3, 25, 2)
	GO:0019885	Antigen processing and presentation of endogenous peptide antigen via MHC class I	3.99*E*− 5	178.99 (6712, 3, 25, 2)
	GO:0019883	Antigen processing and presentation of endogenous antigen	7.96*E*− 5	134.24 (6712, 4, 25, 2)
	GO:0007218	Neuropeptide signaling pathway	1.41*E*− 4	28.77 (6712, 28, 25, 3)

2	GO:0000790	Nuclear chromatin	1.81*E*− 4	9.31 (6712, 68, 53, 5)
	GO:0000785	Chromatin	4.71*E*− 4	5.94 (6712, 128, 53, 6)
	GO:0071778	WINAC complex	6.03*E*− 4	50.66 (6712, 5, 53, 2)
	GO:0016591	DNA-directed RNA polymerase II, and holoenzyme	6.03*E*− 4	50.66 (6712, 5, 53, 2)

3	GO:0005212	Structural constituent of eye lens	7.99*E*− 7	1,118.67 (6712, 4, 3, 2)
